# Comprehensive Characterization of Circular RNAs in Ovary and Testis From Nile Tilapia

**DOI:** 10.3389/fvets.2022.847681

**Published:** 2022-04-06

**Authors:** Huan Zhong, Zhongbao Guo, Jun Xiao, Hong Zhang, Yongju Luo, Junneng Liang

**Affiliations:** ^1^Hunan Research Center of Engineering Technology for Utilization of Distinctive Aquatic Resource, College of Animal Science and Technology, Hunan Agricultural University, Changsha, China; ^2^Guangxi Tilapia Genetic Breeding Center, Guangxi Academy of Fishery Sciences, Nanning, China; ^3^Guangxi Key Laboratory of Beibu Gulf Marine Biodiversity Conservation, Beibu Gulf University, Qinzhou, China

**Keywords:** circRNA, miRNA, gonad, tilapia, sex change

## Abstract

Circular RNA (circRNA) is an endogenous biomolecule in eukaryotes. It has tissue- and cell-specific expression patterns and can act as a microRNA sponge or competitive endogenous RNA. Although circRNA has been found in several species in recent years, the expression profiles in fish gonad are still not fully understood. We detected the expression of circRNA in the ovary, testis, and sex-changed gonad of tilapia by high-throughput deep sequencing, and circRNA-specific computing tools. A total of 20,607 circRNAs were obtained, of which 141 were differentially expressed in the testis and ovary. Among these circRNAs, 135 circRNAs were upregulated and 6 circRNAs were downregulated in female fish. In addition, GO annotation and KEGG pathway analysis of the host genes of circRNAs indicated that these host genes were mainly involved in adherens junction, androgen production, and reproductive development, such as ZP3, PLC, delta 4a, ARHGEF10, and HSD17b3. It is worth noting that we found that circRNAs in tilapia gonads have abundant miRNA-binding sites. Among them, 935 circRNAs have a regulatory effect on miR-212, 856 circRNAs have a regulatory effect on miR-200b-3p, and 529 circRNAs have a regulatory effect on miR-200b-5p. Thus, our findings provide a new evidence for circRNA–miRNA networks in the gonads in tilapia.

## Introduction

Circular RNA (circRNAs) is a class of noncoding RNAs molecules, which do not have 5′ terminal caps and 3′ terminal poly (A) tails (1). It has a covalently connected closed loop structure, and is generated by the event of reverse splicing (backsplice, also known as the first splicing, head-to-tail splice) (2). In unicellular organisms, circRNAs are mainly derived from the self-splicing introns of preribosomal RNA, while in eukaryotes, circRNAs can be derived from the self-splicing introns and exons of pre-mRNA (3–5). By using high-throughput deep-sequencing technology, a large number of circRNAs have been recently found in expressions in human cells, mouse, zebrafish, nematode, and *Larimichthys crocea* (6–11). It acted as a miRNA sponge in cytoplasm, or as an isolator of RNA-binding protein (RBP), or as a regulator of nuclear translation, which suggests that circRNA may play an important role in the gene expression regulation network (3, 12).

One of the main objectives of aquaculture is to improve the reproductive efficiency of fish, which can be achieved through genetic selection between sexes (13). For most vertebrates, during embryonic development, “sex determination” triggers the development of the initial bipotential gonads into ovaries or testes to determine their sex (14–16). The gonad determines its subsequent sexual differentiation and related physiological characteristics (16, 17). Sex of teleost depends on its genetics (such as XY, ZW, and polygene), environment (such as temperature, pH, and population density), behavior, and physiological factors (15, 16). However, a large number of evidences show that teleost has great plasticity in gonadal development and sexual expression, which can start life in one sex and then change to another (16, 18, 19). It includes species that can undergo hermaphroditic (male-to-female or female-to-male) or bidirectional sex change (20). Sex change has existed in many species in nature, which is a natural phenomenon of lower animals in order to reproduce (21, 22). Fish has a wide range of phylogeny and natural sex changes, which makes it a very useful model to study the function and evolution of vertebrate sex determination and differentiation system (16, 23). Up to now, the details about circRNA in fish sex change are still limited. This information will help further understand the identification characteristics of fish gonad or better improve the quality and production of fish.

CircRNA is widely expressed in tissues (24). Compared with the linear RNA molecule, the structure of the circRNAs is more stable, and it can inhibit the degradation of RNase R enzyme (25, 26). Some circRNAs have developmental stage specificity and interspecies conservatism (24, 25). A previous study on circular RNA in mouse testis have found that the sex-determining region Y (SRY) gene is expressed in a circular isoform and can produce linear mRNA in early embryonic development, which plays a key role in sex determination (27). Compared with mammalians, circRNA expression in fish gonad is less. In the present study, we tried to find differentially expressed circRNAs from female, male, and sex-changed tilapia. Then we analyzed the characterization of differentially expressed circRNAs, which may provide a basis for further research of circRNA function in tilapia.

## Materials and Methods

### Ethics Approval

This study was conducted following the Guide for the Care and Use of the Guangxi Academy of Fishery Sciences (Nanning, China). All procedures for fish handling were performed after fish were anesthetized in MS-222 (3-aminobenzoic acid ethyl ester methanesulfonate).

### 17α-Methyltestosterone Treatment

All Nile tilapias were provided by the Guangxi Academy of Fishery Sciences. A total of 30 fish were used in the experiment. To ensure minimal disturbance of the fish during sampling, the fish were fed twice at the same time each day (8:00 and 17:00). The level of intake is 3% of all fish weight. Water parameters were monitored and maintained as follows: temperature at 28.0 ± 1.0°C and dissolved oxygen at 60 ± 5%. The light intensity at the water surface followed the sunlight. The fish were maintained in three breeding tanks (3-m diameter and 1.5-m water depth) and were randomly placed in each tank assigned to male (M), female (F), and sex-changed (R) groups (*n* = 10 for each group). The fish in the M and F groups were fed with commercial tilapia diet, while the fish in the R group were fed with a diet that contained 50 mg of 17α-MT/kg (bodyweight) from 10 to 40 days posthatch (dph) (28). After 40 dph, all the tested fish were fed with a normal diet.

### Sample Collection

After 2 months of feeding post-7α-MT/kg treatment, all the fish were dissected, and the different types of gonads were calculated. Three female, three male fish, and three sex-changed tilapias were collected for circRNA sequencing. All the gonad samples were washed with cold, sterile 1 × PBS to remove contaminating blood before immersion in liquid nitrogen. Then the samples were stored at −80°C until RNA extraction.

### RNA Extraction and Circular RNA Sequencing

The total RNAs were isolated from the collected gonads with TRIzol Reagent (Invitrogen, Carlsbad, CA, USA). The total RNAs were tested by NanoDrop 2000 spectrophotometer (Thermo Scientific, USA) to assay the purity and concentration. The RNase R (RNA07250, Epicenter, USA) to digest the linear RNAs and RNeasy MinElute Cleanup Kit (Qiagen) to purify the RNAs were used.

According to the manufacturer's instructions, a chain-specific library was constructed using Illumina's VAHTS total RNA SEQ (H/M/R) library preparation kit. Ribosomal RNAs were removed to retain circRNAs. The enriched circRNAs were then fragmented, reverse transcribed into cDNA, added poly (A), connected to Illumina sequencing adapter, and finally digested by uracil-N-glycosylase (UNG). After purification, the digested products were amplified and sequenced by Illumina HiSeq 2500.

By excluding low-quality sequences (*Q* ≤ 30 bases) or reads containing more than 3% wrong nucleotides (n), all clean reads were obtained through the sequencing adapter. The short reads alignment tool Bowtie2 was used to compare the clean reads with the ribosomal RNA (rRNA) database. The rRNA removal readings of each sample were mapped to the reference tilapia genome (O_niloticus_UMD_NMBU) (https://www.ncbi.nlm.nih.gov/assembly/GCF_001858045.2/) by TopHat2 (v. 2.0.3.12). Twenty mers were extracted from both ends of the unmapped reading segment and compared according to the reference genome to find a unique anchor position in the splice site. The anchor readings arranged in the opposite direction (from head-to-tail) indicated the splicing of circRNA, and then the Findcirc was performed to identify the circRNA. CircRNAs were blasted with circBase for annotation. At last, the type, chromosome distribution, and length distribution of the identified circRNAs were statistically analyzed.

### Expression Profiling and Analysis of Differentially Expressed Circular RNAs in Three Types of Fishes

In order to quantify the expression of circRNAs, the RPM (reads per million mapped reads) method, which represents the back-spliced junction reads, was used to eliminate the influence of different amounts of sequencing data on the calculation of circRNA expression. The edgeR package (http://www.rproject.org/) was used to identify the differentially expressed circRNA among different samples. In the comparison among the samples, we identified that circRNAs with fold change ≥2 and *p* < 0.05 were differentially expressed.

### Gene Ontology and KEGG Pathway Enrichment Analysis

We performed functional enrichment analysis on the source genes of circRNAs to show their main functions. GO enrichment analysis provides all GO terms that are significantly enriched in the source genes compared with genomic background and filters the source genes corresponding to biological functions. First, all source genes are mapped to GO terms in the Gene Ontology database (http://www.geneontology.org/). Taking FDR ≤ 0.01 as the threshold, the false discovery rate (FDR) was corrected for the calculated *p*-value. The GO terms that meet this condition in the source genes are called the enrichment GO terms.

KEGG pathway analysis is helpful to clarify the biological function of genes. Compared with the genome-wide background, pathway enrichment analysis identified metabolic pathways or signal transduction pathways with significant enrichment in the source genes. The calculated *p*-value also is corrected by FDR, and FDR ≤ 0.05 is the threshold. The pathway satisfying this condition is defined as the pathway of significant enrichment in the source gene.

### MicroRNA Target Site Analysis

For the circRNAs with intersection determined by the three methods, MIREAP (https://sourceforge.net/projects/mireap/), MIRANDA (v. 3.3a) (29), and TARGETSCAN (v. 7.0) (30) were used to predict the microRNA (miRNA) sites. The number of miRNA sites is helpful to identify the circRNA that may act as a miRNA sponge in the tilapia gonad.

## Results

### Overview of Circular RNA Profiles in Male, Female, and Sex-Changed Tilapias

**Figure 1:**
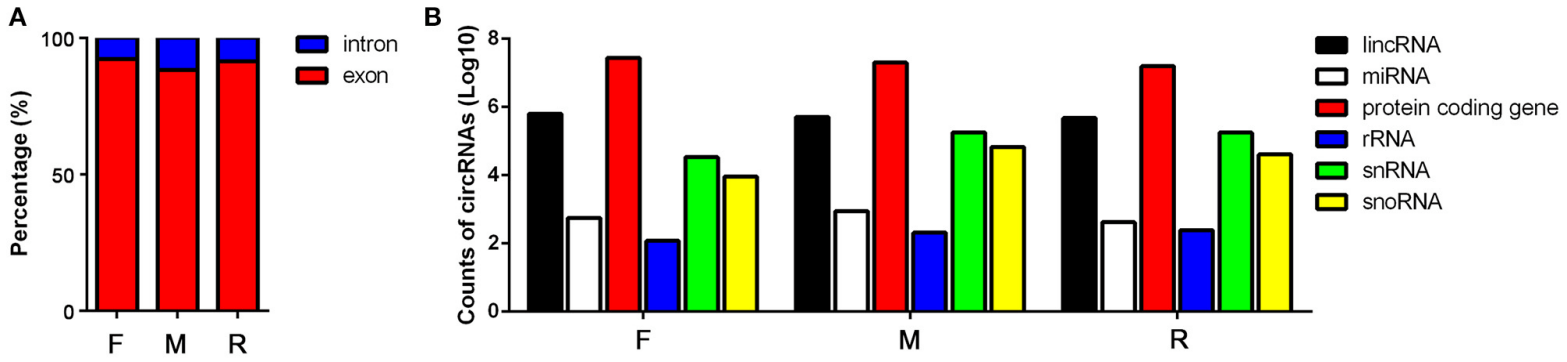
Basic information of the sequencing results from female (F), male (M), and sex-changed (R) tilapias. **(A)** Proportion of the circular RNAs (circRNAs) transcribed from introns and exons from the three groups of tilapias. **(B)** Proportion of the circRNAs transcribed from the lncRNA, microRNA (miRNA), protein coding gene, rRNA, snRNA, and snoRNA regions in the genome.

In order to assay the expression pattern of circRNA during the sex development of tilapia, we profiled circRNAs in the ovaries of female, testis of male, and sex-changed tilapias. After removing the adapter sequences and filtering low-quality reads, a total of 62,554,872, 51,742,312, 56,320,530, 43,308,038, 32,693,730, 41,777,260, 47,670,608, 47,341,848, and 36,952,742 reads (66.74, 73.3, 83.97, 67.59, 82.25, 73.31, 58.65, 83.71, and 80.66% of the total mapped reads) were obtained from the nine samples (Supplementary Table 1). In total, 20,607 candidate circRNAs were identified by at least one read spanning a head-to-tail splice junction. According to their position in the genome, the proportion of exons and introns in the identified circRNAs is also different (Supplementary Table 1). In the male fish, the proportion of introns is the highest (11.76%), and in the female tilapia, the proportion of exons is the highest (92.68%) (Figure 1A). Comparing the positions of circRNAs and their source genes in the transcription reference region, it is obvious that most of the circular RNAs of the three groups are produced by the regions of protein-coding genes (Figure 1B).

### Analysis of Differentially Expressed Circular RNAs in Male, Female, and Sex-Changed Tilapias

**Figure 2:**
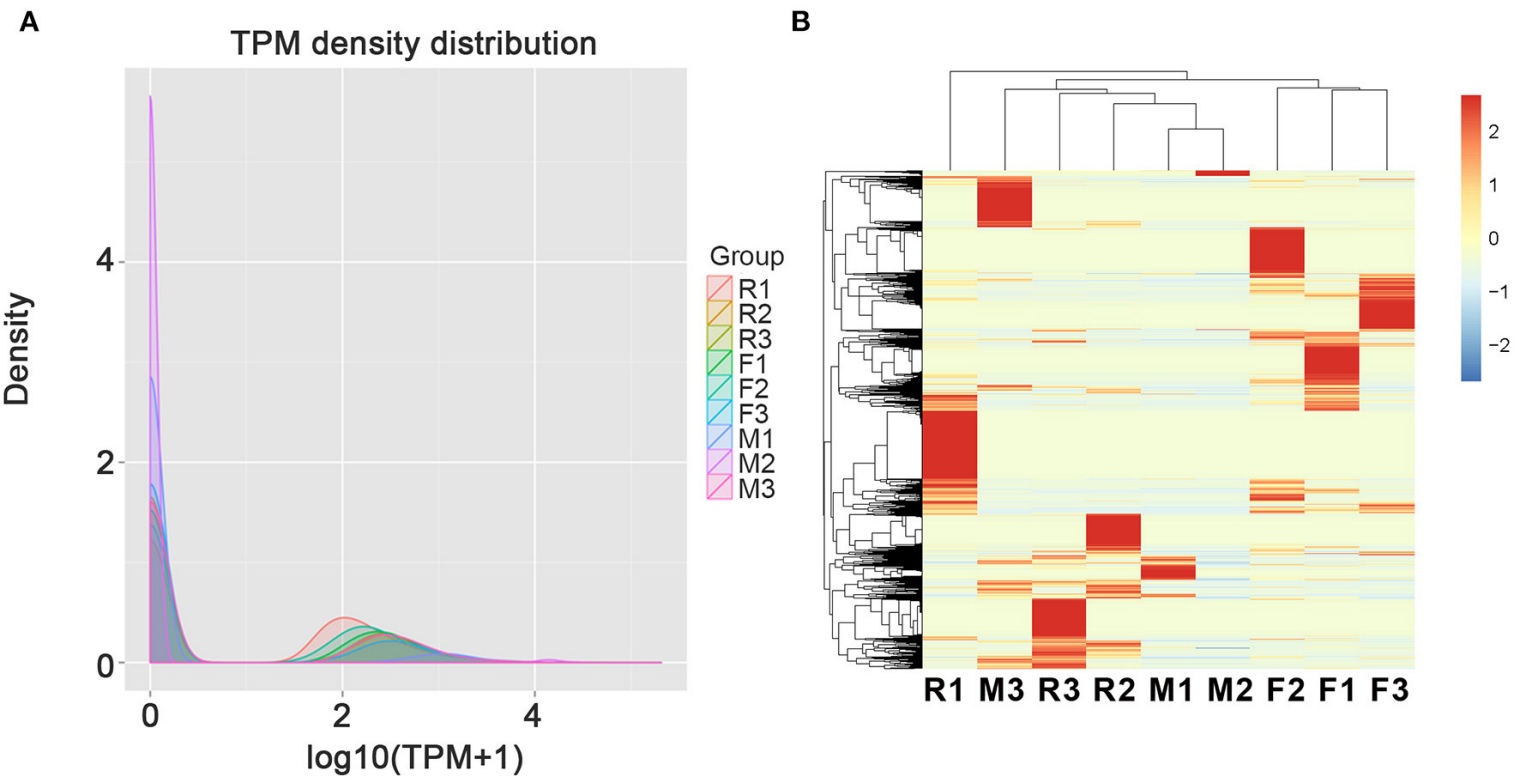
The total expression profile of the three individuals from female (F1–F3), male (M1–M3), and sex-changed (R1–R3) tilapias. **(A)** The expression profiles determined by TPM density of circRNAs in the gonads of female, male, and sex-changed tilapias. **(B)** Heatmap of circRNAs in the gonads of female, male, and sex-changed tilapias.

In order to perform the differentially expressed circRNAs in tilapias, the expression patterns of the identified circRNAs were analyzed. When calculating the expression of different samples, we used TPM to count the differential expression of circular RNA. The distribution of TPM expression levels of all the samples is inconsistent suggesting the expression differences among the types of gonads (Figure 2A). According the heatmap of Figure 2B, the individuals of the F group were clustered together, while the individuals of the M and R groups were clustered together showing higher similarity of the M and R groups. In addition, the expression among individuals of the M and R groups had high diversity of circRNA expression.

**Figure 3:**
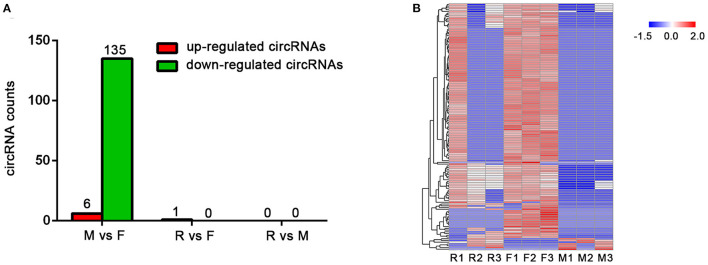
Differentially expressed circRNAs in female (F), male (M), and sex-changed (R) tilapias. **(A)** The counts of upregulated and downregulated circRNAs from the M vs. F, R vs. F, and R vs. M comparisons. **(B)** Heatmap of differentially expressed circRNAs among the groups.

According to the circRNA expression profiles, compared with the female group, the expression of six circRNAs in the male group was upregulated, while the expression of 135 circRNAs was downregulated, which was consistent with the expression of circRNAs in the male group, it was lower than that in the female group (Figure 3A). Interestingly, there was no significant difference between the R group and the other two groups; only one circRNA expression was found to be increased in the R group compared with the female group. The above results illustrate two points: First, because the individual differences between the three samples in the R group are large, there are a few differences in circRNA that can be screened by statistics when compared with the other groups (Figure 3B). Second, the specificity and independence of the R group makes the expression pattern of circular RNA between the male group and female group (Supplementary Table 2).

### Gene Ontology and KEGG Pathway Enrichment Analysis

**Figure 4:**
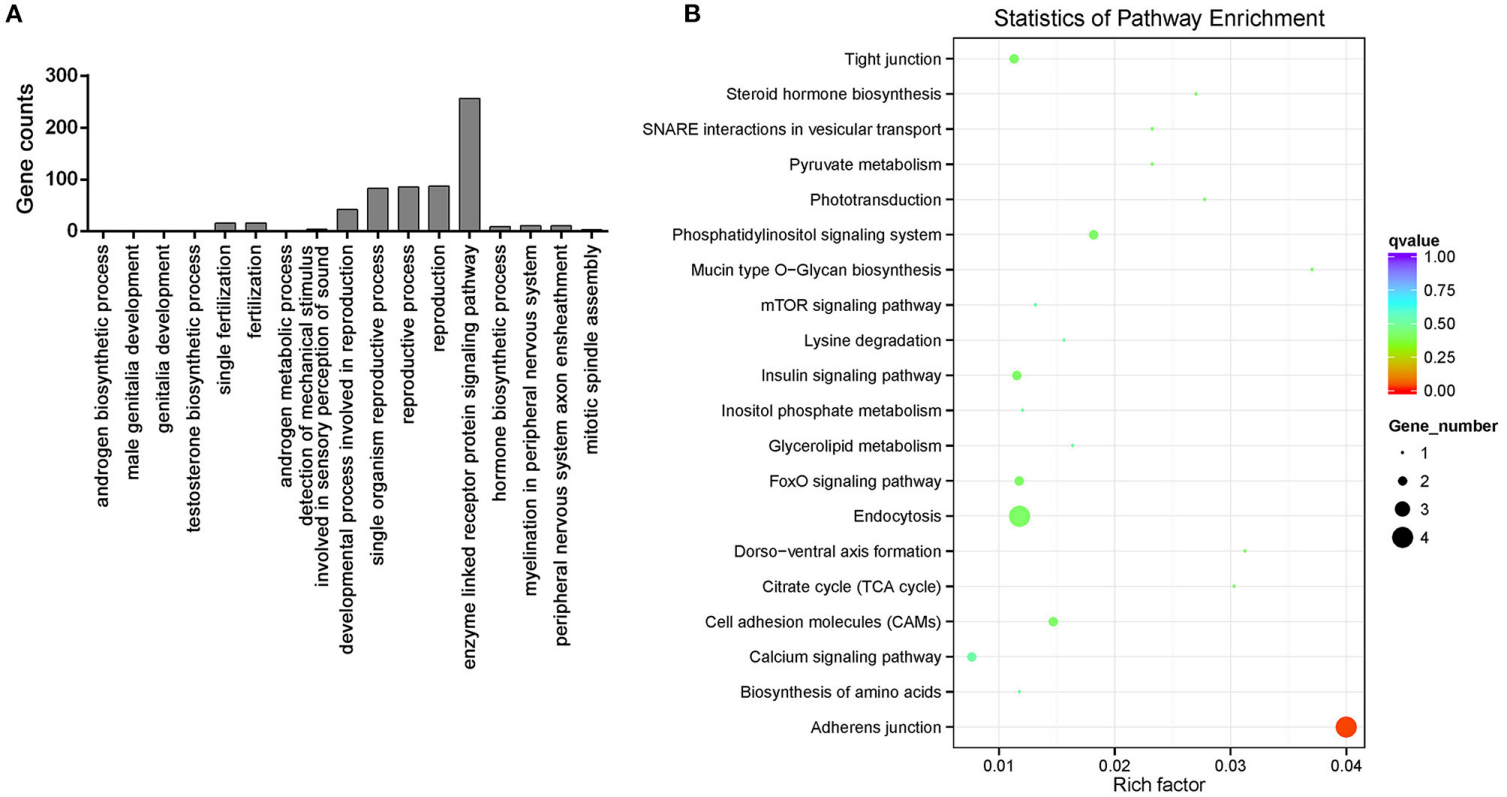
Annotation of the host genes of differentially expressed circRNAs. **(A)** Gene Ontology (GO) annotation analysis of the host genes of differentially expressed circRNAs. **(B)** KEGG pathways of differentially expressed circRNAs host genes.

To explore the putative function of the female and male tilapia circRNAs, GO categories and KEGG pathway analyses were performed on the 20,607 circRNA host genes. The host gene function of the differentially expressed circRNAs between the female and male groups was shown by GO term analysis. The GO term of significant enrichment is listed in Supplementary Table 3. GO annotation analysis showed that the circRNA host genes were involved in androgenesis, reproductive development, and many other GO terms, such as the differentially expressed circRNA host gene zona pellucida sperm-binding protein 3 (ZP3), phospholipase C (PLC), delta 4a, rho guanine nucleotide exchange factor 10 (ARHGEF10), and hydroxysteroid 17-beta dehydrogenase 3 (HSD17b3) (Figure 4A; Supplementary Table 3).

Based on the KEGG pathway database, a pathway analysis was conducted to predict the metabolic and signal transduction pathways, which is significantly enriched in differentially expressed circRNA host genes. The KEGG pathway analysis showed that the circRNA host genes are only significantly enriched in adherens junction pathway (*p* < 0.05) (Figure 4B).

### Prediction of MicroRNA Target Sites in Circular RNAs of Tilapia

**Figure 5:**
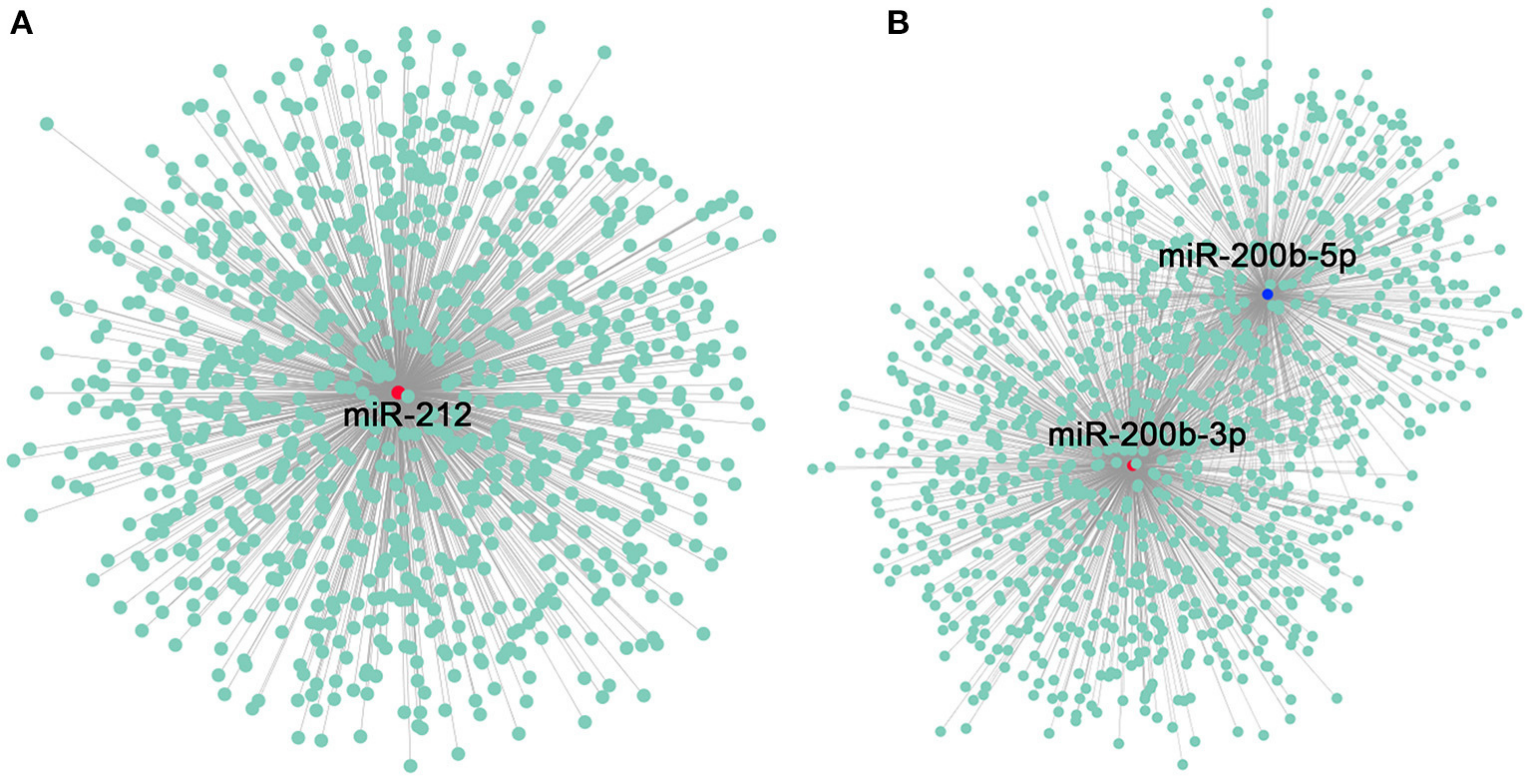
Construction of miRNA-212–circRNA and miR-200b–circRNA networks. **(A)** miRNA-212–circRNA network constructed using Cytoscape. The red dot indicates miR-212, and the green dots indicate the related circRNAs. **(B)** miR-200b–circRNA network constructed using Cytoscape. The red dot indicates miR-200b-3p, the blue dot indicates miR-200b-5p, and the green dots indicates the circRNAs as miRNA sponges.

CircRNAs can act as miRNA sponges and bind to miRNAs to inhibit their targeted mRNAs, thereby regulating upstream gene expression. In order to reveal whether circRNAs in the gonad of tilapia can target miRNAs and further influence the posttranscriptional regulation of gonadal genes, we used circRNA sequence to identify the potential binding sites of miRNAs. miRNA prediction was performed *via* the combination of software, and it shows that a total of 8,550 circRNAs can regulate 350 miRNAs (Supplementary Table 4). Because miR-212 and miR-200b have been proven to be involved in sex differentiation of tilapia in previous studies, we used these two miRNAs as binding sites in this study, and the results suggested that a total of 935 circRNAs can regulate miR-212 (Supplementary Table 5), 856 circRNAs can regulate miR-200b-3p, and 529 circRNAs can regulate miR-200b-5p (Supplementary Table 6; Figure 5).

## Discussion

In this study, we demonstrated the circular RNA in tilapia gonads and identified the circRNAs in sex-changed tilapia. Using high-throughput in-depth sequencing, we identified 20,607 circRNAs in the tilapia gonads. Considering the individual differences in the methods of identifying circRNAs in fish transcriptome data analysis, we combined a variety of algorithms to identify possible circRNAs. In fact, the identified expression patterns of circRNAs show that there are significant differences between individuals. Meanwhile, the expression pattern of circRNAs in sex-changed tilapia was almost the same as that in the M and R groups.

The formation of circRNA results from the splice of pre-mRNA, and the different splice sites lead to different circRNAs produced by exons and introns of genes (3, 25). circRNAs are mainly produced by exon circularization, and most of these circRNAs are produced by the expression of known protein-coding genes that consist of one exon or multiple exons (3, 26, 31). Previous reports found that introns control the rate of circular RNA production, but the retained part does not exceed 20% (31, 32). This has also been confirmed in our research that due to the unique splicing method of circRNA, the proportion of exons in circRNA sequences in tilapias exceeds 80%, and the proportion of introns is <20%.

More and more studies on ovary or testis transcriptomes using RNA-Seq have been conducted (33–35). A few studies have focused on the expression profile of circRNA in gonads. In order to further define the differences of circRNAs in sex differentiation, we determined the expression enrichment of circRNAs in male, female, and sex-changed tilapia, and found that 135 circRNAs were downregulated and only 6 were upregulated in the male tilapia compared with the female tilapia. Meanwhile, there was less difference in the expression of circRNAs between the R group and other groups. In the RNA sequences of the zebrafish brain, the differentially expressed lncRNAs in females had higher expression than that in males, and lncRNAs–mRNA coupling in the coexpression network enriches the “hormone response” (33). In goldfish, treatment with testosterone or estrogen did not increase the level of GtH (18, 36). In tilapia, dopamine also inhibited the release of GtH (LH) from the pituitary cells, but did not affect the mRNA level of the gene, suggesting that the upstream gene or hormone did not play a role (18, 37). Because of the variability of fish sex affected by the external environment, we speculate that the reason why sex-changed fish is not different from the other two groups may be because of the instability of the fish sex determination system.

Our GO annotation and KEGG pathway analyses of circRNA host genes between different sexes of tilapia further clarify the biological processes involved in circRNAs. In GO terms, multiple host genes were assigned to androgen production and reproductive development, including ZP3 and HSD17b3 (38, 39). ZP3 promotes the combination of sperm and egg envelope and induces acrosome reaction (38). 17β-hydroxysteroid dehydrogenase (17βHSD) regulates the levels of active androgens and estrogen in a tissue-specific manner, and when the 17βHSD3 gene is mutated, it can cause male pseudohermaphroditism (40, 41). In our study, ZP3 and HSD17b3 are involved in tilapia androgen production and reproductive development, and are host genes for differential expression of circRNA. In the reports of mice, humans, and fish, the expression of these two genes is also significantly higher in the ovaries than in the testes (38, 39). In the KEGG pathway, 20 pathways were significantly enriched, and only adherens junctions were significantly enriched (*p* < 0.05). Cell–cell actin-based adhesion junctions and desmosomes mediated by cell–cell intermediate membrane are the cell adherens junctions' modes between spermatogenic epithelium (42). In the zebrafish, yellow catfish, spotted scat, and dark sleeper, hormonal genes are also significantly enriched from differentially expressed genes in different types of gonads (33, 34, 43, 44). Thus, the present result is similar to the previous studies.

A circRNA can serve as an miRNA sponge for multiple different miRNAs instead of containing multiple sites of a specific miRNA, thereby, having multiple functions (24). At the same time, because of the relationship between the miRNA-binding sites of circRNA and the mRNA target sites of miRNA, the high abundance of circRNA with many competitive binding sites can bind to more miRNAs (24, 45, 46). In this study, miRNA-212 was matched with 935 circRNAs, miRNA-200b-3p was matched with 856 circRNAs, and miRNA-200b-5p was matched with 529 circRNAs, which were reported in the study of tilapia sex differentiation; miRNA-212 and miRNA-200 played important roles (47, 48). Since overexpression of miRNA target site concatamers (miRNA sponges) can lead to loss of miRNA function and increased expression of endogenous target genes (12), we speculate that related circRNAs in tilapia may be used as miRNA sponges to control sexual differentiation.

## Conclusion

This is the first time to study circRNAs in female, male, and sex-changed tilapias, revealing the genomic characteristics and differential expression of circRNAs, which lays a foundation for further study on the function of circRNAs in the gonads of tilapia. These findings provide a new strategic approach for the identification and characterization of the key circRNA–miRNA networks in sexual differentiation.

## Data Availability Statement

The datasets presented in this study can be found in online repositories. The names of the repository/repositories and accession number(s) can be found in the article/Supplementary Material.

## Ethics Statement

The animal study was reviewed and approved by Care and Use of Guangxi Academy of Fishery Sciences.

## Author Contributions

HZho, JX, and YL conceived and designed the study. HZha, ZG, and JL collected the samples. HZho analyzed the data. HZho and ZG wrote the manuscript. All authors have read and approved the final version of the manuscript.

## Funding

This study was supported by the National Natural Science Foundation of China (31760756), the Natural Science Foundation of Guangxi (2016GXNSFFA380002 and 2017GXNSFFA198001), the Guangxi Key Laboratory of Beibu Gulf Marine Biodiversity Conservation (2020KB02), the National Key Research and Development Program of China (2018YFD0900601), and the China Agriculture Research System (CARS-46).

## Conflict of Interest

The authors declare that the research was conducted in the absence of any commercial or financial relationships that could be construed as a potential conflict of interest.

## Publisher's Note

All claims expressed in this article are solely those of the authors and do not necessarily represent those of their affiliated organizations, or those of the publisher, the editors and the reviewers. Any product that may be evaluated in this article, or claim that may be made by its manufacturer, is not guaranteed or endorsed by the publisher.
